# HIV testing among transgender and nonbinary persons in Michigan, United States: results of a community‐based survey

**DOI:** 10.1002/jia2.25972

**Published:** 2022-10-12

**Authors:** Ashley Lacombe‐Duncan, Leonardo Kattari, Shanna K. Kattari, Ayden I. Scheim, Flyn Alexander, Sophie Yonce, Brayden A. Misiolek

**Affiliations:** ^1^ School of Social Work, University of Michigan Ann Arbor Michigan USA; ^2^ School of Social Work, Michigan State University East Lansing Michigan USA; ^3^ Department of Women's and Gender Studies, University of Michigan Ann Arbor Michigan USA; ^4^ Dornsife School of Public Health, Drexel University Philadelphia Pennsylvania USA; ^5^ Lyman Briggs College, Michigan State University East Lansing Michigan USA; ^6^ Transcend the Binary Ferndale Michigan USA

**Keywords:** HIV care cascade, transmasculine, transfeminine, gender diverse, stigma, gender affirmation

## Abstract

**Introduction:**

Transgender (trans) and nonbinary people (TNB) are disproportionately impacted by HIV. HIV testing is critical to engage TNB people in HIV prevention and care. Yet, scant literature has examined social and structural factors associated with HIV testing among TNB people of diverse genders and in geographies with potentially lower trans acceptance. We: (1) characterized the prevalence of never having been tested for HIV; and (2) identified associated factors, among TNB people in Michigan, United States.

**Methods:**

Data were from a community‐based participatory cross‐sectional survey (*n =* 539 sexually experienced TNB people). The prevalence of never having had an HIV test was reported overall and compared across socio‐demographic, clinical, social and structural factors using bivariable and multivariable logistic regression analyses.

**Results and discussion:**

Approximately one‐quarter (26.2%) of participants had never had an HIV test (20.8% transfeminine; 30.0% transmasculine; 17.8% nonbinary assigned male at‐birth; and 32.0% nonbinary assigned female at‐birth). In a multivariable socio‐demographic model, older age (adjusted odds ratio [aOR] for 1‐year increase: 0.93, 95% CI: 0.90, 0.96, *p<*0.001) and Black/African American race (vs. White) (aOR: 0.28, 95% CI: 0.09, 0.86, *p<*0.05) were associated with increased odds of HIV testing (aORs for never testing). In separate multivariable models controlling for socio‐demographics, ever experiencing sexual violence (aOR: 0.38, 95% CI: 0.21, 0.67, *p<*0.001), not accessed sexual/reproductive healthcare in the past 12 months (aOR: 4.46, 95% CI: 2.68, 7.43, *p*<0.001) and reporting a very/somewhat inclusive primary care provider (PCP) (aOR: 0.29, 95% CI: 0.17, 0.49, *p<*0.001) were associated with HIV testing (aORs for never testing).

**Conclusions:**

Findings contribute to scant literature about gender‐based differences in HIV testing inclusive of transmasculine and nonbinary people. Lack of statistically significant gender differences suggests that broad TNB interventions may be warranted. These could include training healthcare providers in trans‐inclusive practices with sexual violence survivors and PCPs in trans‐inclusive HIV prevention and care. Findings showing Black participants were less likely to have never had an HIV test suggest the promise of culturally tailored services, though further investigation is needed. Findings identify social and structural factors associated with HIV testing and can inform multi‐level interventions to increase TNB person's HIV testing.

## 1 INTRODUCTION

Transgender (trans) and nonbinary (TNB) people are disproportionately impacted by HIV [1, 2]. HIV testing is a critical first step to engage TNB people in HIV prevention and care. Yet, research has identified HIV care disparities among trans women compared to cisgender (cis) persons [3, 4, 5, 6, 7, 8, 9, 10], including lower HIV testing rates [11]. Findings from a national probability sample of sexually active trans people in the United States reported that while nearly half of respondents (46.4%) met Centers for Disease Control and Prevention (CDC) recommendations for HIV testing, almost one‐quarter (22.8%) had never tested for HIV, identifying no significant differences between trans women and trans men [12]. A paucity of literature has examined within‐TNB community differences or HIV testing among nonbinary persons, who comprise one‐third of U.S. trans people [13].

There are also gaps in understanding multi‐level factors associated with HIV testing among TNB persons, particularly anti‐trans stigma and gender affirmation. Quantitative studies have shown negative associations between anti‐trans stigma and HIV care access [14], whereas qualitative studies have identified how intersecting anti‐trans and HIV stigma limit trans women's access to HIV prevention/care [15]. Conversely, gender affirmation, the process of recognizing and supporting a TNB person's gender, is associated with engagement in HIV care and viral suppression [16] and uptake of biomedical HIV prevention [12, 17].

Finally, much U.S. TNB‐focused HIV testing research has been conducted in large urban centres with higher trans acceptance (e.g. New York City [18, 19]), limiting our understanding of HIV testing among TNB persons in other, potentially more stigmatizing, areas of the United States, such as Michigan, part of the U.S. Midwest [20]. Michigan's population is just under 10 million [21] with 18,970 persons living with HIV [22]. New diagnoses are primarily concentrated in Detroit, among the most racially segregated U.S. regions [23, 24]. Michigan has limited protections for lesbian, gay, bisexual, trans and queer (LGBTQ+) people [25], with lower LGBTQ+ equality than several states [26]. For example, both a national LGBTQ+ youth survey (23.1% Midwest) [27] and a qualitative study with Midwestern TNB youth [28, 29] identified pervasive interpersonal and structural sexual and anti‐trans stigma and negative impacts on participants’ wellbeing.

The aims of this study were to: (1) characterize the prevalence of never testing for HIV, and (2) identify associated socio‐demographic, clinical, social and structural factors, among trans and nonbinary people in Michigan, United States.

## 2 METHODS

### 2.1 Study design

This study utilized secondary data from the Michigan Trans Health Survey (MTHS) [30], an online survey with 659 TNB people (2018). Survey items were collected from study investigators, a TNB advocacy group and TNB people [30]. Eligible participants were those 18 years of age or older, living in Michigan and identifying as transgender, trans, nonbinary, genderqueer, agender, genderfluid, two‐spirit, transsexual or another non‐cisgender identity. The sample for this paper was limited to those self‐reporting having ever been sexually active (yes/no). As described elsewhere [30, 31], participants were recruited using convenience methods both online (e.g. Facebook) and in‐person (e.g. Pride events) as well as snowball sampling. The survey was determined exempt by the University of Michigan Institutional Review Board (HUM00143266). All participants clicked a box to indicate informed consent prior to beginning the survey. Participants were provided a $10 USD gift card upon completion of the survey.

### 2.2 Measures

The primary outcome of lifetime HIV testing history was assessed by asking “When was the last time you took an HIV test” with response options: “within the last year,” “more than 1 year ago but less than three years ago,” “three to five years ago” and “more than five years ago” (categorized as ever) versus “I have never taken an HIV test” categorized as never.

Socio‐demographic factors included age, gender identity (transfeminine; transmasculine; nonbinary assigned male at‐birth [AMAB]; nonbinary assigned female at‐birth [AFAB]); sexual orientation (monosexual [i.e. attracted to one gender]; heterosexual; monosexual sexual minority; asexual/demisexual [i.e. primarily nonsexual attraction]; polysexual [i.e. attracted to multiple genders]), race/ethnicity (Black/African American; Latinx/Chicanx/Hispanic; multiracial/biracial; additional races; White), one or more disabilities (yes; no), geographic locality (small city [10,000 to <100,000 people]/rural [<10,000 people]/frontier [< 6 people/square mile]; urban [cities ≥ 100,000 people]/suburban [neighbourhoods on outskirts/near cities ≥100,000 people]), education (high school/GED or less; some college; trade school/associates degree; bachelors degree; graduate degree) and relationship status (single/divorced; casually dating; multiple committed partners; one committed partner). Clinical factors included self‐reported current use of gender‐affirming hormones via any source (e.g. prescription/non‐prescription) (yes/no) and illicit substance use (yes/no). Social/structural factors included lifetime sexual violence (yes/no), past 12‐month experience of discrimination based on gender identity when accessing sexual or reproductive healthcare (yes/no/did not access this care in the past 12 months), trans inclusivity of ones’ primary care provider (PCP) (does not have a PCP/neutral or not inclusive/very or somewhat inclusive) and health insurance type (private [self/partner], private [parent(s)], public).

### 2.3 Analyses

Descriptive statistics were analysed for all variables overall and by HIV testing history (ever vs. never). Then, we estimated unadjusted associations between socio‐demographic, clinical, social and structural factors, and never‐testing for HIV using bivariable logistic regression. Next, we fit a multivariable logistic regression model, including all socio‐demographic variables, to identify those independently associated with never‐testing. Finally, we conducted multivariable analyses whereby each clinical, social and structural factor was examined adjusting for socio‐demographic factors associated with never‐testing at *p<*0.2 (age, gender identity, race, sexual orientation, geographic location and education). All analyses were conducted on cases with complete data as little data were missing (7/14 variables missing no data; range of missing data from 0.4% to 11.5%).

## 3 RESULTS AND DISCUSSION

Among 539 eligible participants, approximately one‐quarter (26.5%, *n* = 143) had never had an HIV test. Among those for whom gender identity data were categorizable (*n* = 521), 21.0% (*n* = 22) of transfeminine participants; 30.7% (*n* = 47) of transmasculine participants; 17.8% (*n* = 16) of nonbinary AMAB participants; and 32.4% (*n* = 56) of nonbinary AFAB participants had never had an HIV test. Almost half of participants (46.2%) had tested < 1 year ago, 13.4% had tested 1 year to < 3 years ago, 6.4% had tested 3 years to < 5 years ago and 7.1% 5 or more years ago (Table 1).

**Table 1:** Socio‐demographic, clinical, social and structural factors by HIV testing history among sexually active trans and nonbinary people in the Midwestern United States (*n* = 539)

	Total sample (*N* = 539)	HIV tested ever (*n* = 396)	HIV tested never (*n* = 143)
Variable	*n* (%) or mean (SD)	*n* (%) or mean (SD)	*n* (%) or mean (SD)
**Socio‐demographic factors**			
Age***	28.8 (9.7)	30.1 (9.9)	25.1 (8.2)
Gender identity (*n* = 521)*			
Transfeminine	105 (20.2)	83 (79.0)	22 (21.0)
Transmasculine	153 (29.4)	106 (69.3)	47 (30.7)
Nonbinary AMAB	90 (17.3)	74 (82.2)	16 (17.8)
Nonbinary AFAB	175 (33.2)	117 (67.6)	56 (32.4)
Sexual orientation (*n* = 514)			
Monosexual (heterosexual)	64 (12.5)	43 (67.2)	21 (32.8)
Monosexual (sexual minority)	112 (21.8)	90 (80.4)	22 (19.6)
Asexual/demisexual	19 (3.7)	12 (63.2)	7 (36.8)
Polysexual	319 (62.1)	228 (71.7)	91 (28.5)
Race (*n* = 515)			
Black/African American	37 (7.2)	33 (89.2)	4 (10.8)
Latinx/Chicanx/Hispanic	23 (4.5)	16 (69.6)	7 (30.4)
Multiracial/biracial	30 (5.8)	23 (76.7)	7 (23.3)
Additional races	18 (3.5)	11 (61.1)	7 (38.9)
White	407 (79.0)	290 (71.3)	142 (28.7)
One or more disabilities			
Yes	239 (43.8)	168 (71.5)	67 (28.5)
No	307 (56.2)	228 (75.0)	76 (25.0)
Geographic locality			
Small city/rural/frontier	164 (30.0)	110 (67.9)	52 (32.1)
Urban/suburban	382 (70.0)	286 (75.9)	91 (24.1)
Education			
High school/GED or less	83 (15.2)	52 (62.7)	31 (37.3)
Some college	189 (34.6)	134 (72.4)	51 (27.6)
Trade school/associates degree	87 (15.9)	69 (79.3)	18 (20.7)
Bachelors degree	122 (22.3)	86 (71.1)	35 (28.9)
Graduate degree	65 (11.9)	55 (87.3)	8 (12.7)
Relationship status			
Single/divorced	150 (27.5)	104 (70.3)	44 (29.7)
Casually dating	47 (8.6)	35 (76.1)	11 (23.9)
Multiple committed partners	44 (8.1)	34 (77.3)	10 (22.7)
One committed partner	305 (55.9)	223 (74.1)	301 (25.9)
**Clinical, social and structural factors**			
Current hormone use (*n* = 537)			
Yes	271 (50.5)	208 (76.8)	63 (23.2)
No	266 (49.5)	187 (70.3)	79 (29.7)
Current illicit drug use (*n* = 523)			
Yes	62 (11.9)	47 (75.8)	15 (24.2)
No	461 (88.1)	340 (73.8)	121 (26.2)
Sexual violence (*n* = 515)***			
Ever	155 (30.1)	132 (85.2)	23 (14.8)
Never	360 (69.9)	249 (69.2)	111 (30.8)
Discrimination in sexual health or reproductive care in the past 12 months (*n* = 528)***			
Yes	86 (16.3)	76 (88.4)	10 (11.6)
No	216 (40.9)	181 (83.8)	35 (16.2)
I did not access this care past 12 months	226 (42.8)	131 (58.0)	95 (42.0)
Trans inclusivity of primary care provider (PCP) (*n =* 537)***			
Does not have PCP	142 (26.4)	85 (59.9)	57 (40.1)
Neutral or not inclusive	112 (20.9)	75 (67.0)	37 (33.0)
Very or somewhat inclusive	283 (52.7)	234 (82.7)	49 (17.3)
Health insurance type (*n* = 477)***			
Private (self/partner)	165 (34.6)	128 (79.5)	33 (20.5)
Private (parent[s])	154 (32.3)	94 (61.0)	60 (39.0)
Public^a^	158 (33.1)	126 (80.3)	31 (19.7)

Note: *n* = 539 unless otherwise specified.

Abbreviations: AFAB, assigned female at birth; AMAB, assigned male at birth; SD, standard deviation.

^a^ Monosexual (i.e. attracted to one gender); asexual/demisexual (i.e. primarily non‐sexual attraction); and polysexual (i.e. attracted to multiple genders).

^*^ *p<*0.05; ^***^ *p<*0.001; analyses conducted using *t*‐test for age and chi‐square for all other variables.

The following socio‐demographic factors were associated with never having had an HIV test in bivariable analyses: age (odds ratio [OR] for 1‐year increase: 0.93, 95% CI: 0.90, 0.95, *p<*0.001), nonbinary AFAB gender (vs. transfeminine) (OR: 1.81, 95% CI: 1.02, 3.19, *p<*0.05), Black/African American race (vs. White) (OR: 0.30, 95% CI: 0.10, 0.87, *p<*0.05) and high school/GED or less, some college, or bachelor's (vs. graduate degree) (OR: 4.10, 95% CI: 1.73, 9.93, *p<*0.01; OR: 2.62, 95% CI: 1.17, 5.59, *p<*0.05; OR: 2.80, 95% CI: 1.21, 6.48, *p<*0.05, respectively) (Table 2). In multivariable analyses, age (adjusted odds ratio [aOR]: 0.93, 95% CI: 0.90, 0.96) and Black/African American race (vs. White) (aOR: 0.28, 95% CI: 0.09, 0.86, *p<*0.05) maintained significance.

**Table 2:** Logistic regression results for socio‐demographic factors in association with never having had an HIV test among trans and nonbinary persons in the Midwestern United States (*n* = 504 adjusted model)

Variables	Unadjusted odds ratio (OR)	95% CI	Adjusted odds ratio (aOR)	95% CI
**Socio‐demographic factors**				
Age	0.93	0.90, 0.95***	0.93	0.90, 0.96***
Gender identity				
Transfeminine (ref)				
Transmasculine	1.67	0.94, 2.99	1.10	0.56, 2.19
Nonbinary AMAB	0.82	0.40, 1.67	0.73	0.32, 1.63
Nonbinary AFAB	1.81	1.02, 3.19*	1.36	0.69, 2.67
Sexual orientation				
Polysexual (ref)				
Monosexual (sexual minority)	0.61	0.36, 1.04	0.80	0.44, 1.45
Monosexual (heterosexual)	1.22	0.69, 2.18	1.54	0.76, 3.14
Asexual/demisexual	1.46	0.40, 3.83	1.63	0.56, 4.74
Race				
White (ref)				
Black/African American	0.30	0.10, 0.87*	0.28	0.09, 0.86*
Latinx/Chicanx/Hispanic	1.08	0.44, 2.70	1.04	0.39, 2.79
Multiracial/biracial	0.75	0.32, 1.81	0.68	0.27, 1.73
Additional races	1.58	0.60, 4.17	1.20	0.41, 3.56
One or more disabilities				
No (ref)				
Yes	1.20	0.82, 1.76	1.02	0.65, 1.61
Geographic locality				
Urban/suburban (ref)				
Small city/rural/frontier	1.49	0.99, 2.23	1.24	0.78, 1.97
Education				
High school/GED or less	4.10	1.73, 9.73**	2.15	0.79, 5.87
Some college	2.62	1.17, 5.59*	1.40	0.55, 3.54
Trade school/associates degree	1.79	0.73, 4.43	1.42	0.53, 3.81
Bachelors degree	2.80	1.21, 6.48*	1.83	0.73, 4.59
Graduate degree (ref)				
Relationship status				
Single/divorced (ref)				
Casually dating	0.74	0.35, 1.59	0.60	0.26, 1.38
One committed partner	0.83	0.53, 1.28	0.75	0.46, 1.25
Multiple committed partners^a^	0.70	0.32, 1.53	0.60	0.25, 1.46

Abbreviations: AFAB, assigned female at birth; AMAB, assigned male at birth; CI, confidence interval.

^a^ Monosexual (i.e. attracted to one gender); asexual/demisexual (i.e. primarily non‐sexual attraction); and polysexual (i.e. attracted to multiple genders).

^*^ *p*<0.05; ^**^ *p*<0.01; ^***^ *p*<0.001.

The following were also statistically significantly associated with never having had an HIV test: ever experienced sexual violence (vs. never) (OR: 0.39, 95% CI: 0.24, 0.64, *p<*0.001), not having accessed sexual or reproductive healthcare in the past 12 months (vs. no discrimination in sexual healthcare or reproductive healthcare in the past 12 months) (OR: 3.76, 95% CI: 2.40, 5.87, *p<*0.001), reporting a very/somewhat inclusive PCP (vs. no PCP) (OR: 0.31, 95% CI: 0.20, 0.49, *p<*0.001) and private insurance, parents (vs. public) (OR: 2.59, 95% CI: 1.56, 4.32, *p<*0.001) (Table 3).

**Table 3:** Logistic regression for clinical, social and structural factors associated with never having had an HIV test among trans and nonbinary persons in the Midwestern United States

Variable	Unadjusted OR	95% CI	Adjusted OR^a^	95% CI
Current hormone use				
No (ref)				
Yes	0.72	0.49, 1.05	0.69	0.41, 1.15
Current illicit drug use				
No (ref)				
Yes	0.73	0.90, 1.66	1.22	0.58, 2.55
Sexual violence				
Never (ref)				
Ever	0.39	0.24, 0.64***	0.38	0.21, 0.67***
Discrimination in sexual health or reproductive care in the past 12 months				
No (ref)				
Yes	0.68	0.32, 1.44	088	0.38, 2.02
Did not access past 12 months	3.76	2.40, 5.87***	4.46	2.68, 7.43***
Trans inclusivity of primary care provider (PCP)				
Does not have PCP (ref)				
Neutral or not inclusive	0.74	0.44, 1.23	0.62	0.34, 1.10
Very or somewhat inclusive	0.31	0.20, 0.49***	0.29	0.17, 0.49***
Health insurance				
Public (ref)				
Private (self/partner)	1.05	0.61, 1.81	0.90	0.48, 1.71
Private (parent[s])	2.59	1.56, 4.32***	1.75	0.95, 3.20

^a^ Adjusting for statistically significant (*p*<0.2) socio‐demographic factors (age, gender identity, race, sexual orientation, geographic location and education).

^***^ *p*<0.001.

Abbreviations: CI, confidence interval; OR, odds ratio.

In multivariable analyses adjusting for socio‐demographic characteristics (age, gender identity, race, sexual orientation, geographic location and education), ever experiencing sexual violence (aOR: 0.38, 95% CI: 0.21, 0.67, *p<*0.001), not having accessed sexual or reproductive healthcare in the past 12 months (aOR: 4.46, 95% CI: 2.68, 7.43, *p*<0.001) and reporting a very/somewhat inclusive PCP (aOR: 0.29, 95% CI: 0.17, 0.49, *p<*0.001) were significantly associated.

While our findings are not contextualized with details regarding sexual or other risks, that almost one‐quarter of sexually active TNB participants had never been tested for HIV warrants further attention given the U.S. CDC recommendation that everyone ages 13–64 be tested for HIV once in their lifetime. However, high HIV testing rates are promising. It could be that national attention to HIV disparities among TNB communities has led to increased awareness of HIV testing needs among this group or that Michigan is more trans‐accepting than hypothesized [32]. We contribute to scant literature about gender‐based differences in HIV testing within TNB communities, finding no statistically significant differences across genders.

Black participants were more likely to have been tested for HIV than their White peers, corroborating other research [12]. Michigan is among the most racially segregated U.S. states, contributing to both health inequities [23] and access to culturally tailored programming (e.g. Trans Sistas of Color Project) [33, 34, 35]. More HIV testing among Black participants may be due to tailored programming or because these participants are more likely to live in Detroit with better access to services. Given racial HIV‐related disparities in the United States [36], these findings suggest that at‐risk populations may be being appropriately tested.

Prior literature found that in addition to disclosure concerns related to being on a parent's insurance posing a barrier to TNB young adults’ access to gender‐affirming healthcare [28], so too is this a barrier to accessing HIV prevention and testing [37, 38]. Options such as free and confidential testing through HIV community‐based organizations (e.g. Unified HIV Health and Beyond [39]) should be discussed with young adults.

Our finding that having a very or somewhat inclusive PCP was associated with increased odds of having ever had an HIV test adds to a growing body of literature about the importance of trans‐inclusive PCPs [40], including qualitative findings with TNB youth from the U.S. Midwest [29]. These findings demonstrate training PCPs to be more trans‐affirming and intersectionally inclusive utilizing evidence‐informed interventions is essential (e.g. [15, 41, 42]). Moreover, findings suggest the need for trans‐affirming support for TNB survivors of sexual violence, potentially fostered through integrating components of promising provider‐level interventions [43, 44] into training more broadly focused on HIV prevention and care for TNB persons, with the potential benefit of increasing access to HIV care [45].

These study results must be interpreted with caution. Cross‐sectional studies do not show causality. Our sample was close to four‐fifths (79%) White, and while representative of Michigan (78% White) [46], a larger sample size of various racial groups would allow us to draw conclusions more relevant to those most affected by HIV in the United States [47] and Michigan [24]. Future studies should track how participants were recruited, as each of recruitment strategy may differentially introduce biases (e.g. online venues may contribute to oversampling of higher socioeconomic status participants) [48]. Our recruitment through TNB‐specific Facebook groups and Pride events may have limited access to those less connected to TNB communities with hypothetically more barriers. As the MTHS, to our knowledge, was the first state‐wide survey conducted with TNB people, we are unable to conclusively determine the extent to which our sample represents the broader TNB population. However, drawing on the data reported in the state‐specific U.S. Transgender Survey (USTS) report (*N =* 894 Michigan participants) [49], metrics such as lifetime homelessness (34% USTS; 39% MTHS) and past‐year anti‐trans discrimination in healthcare (38% USTS; 28% MTHS) [49] were similar. These comparisons lend confidence to the representativeness of our sample.

While we cannot say for certain participant's sexual and other risk practices (e.g. illicit substance use) warranted ongoing or recent HIV testing, given the CDC recommendation, the expectation is that all participants should have been tested for HIV at least once in their lifetime. TNB community partners explicitly requested the removal of a standardized question about sexual risk practices on the MTHS, which they saw as problematic (e.g. assuming condomless anal sex is a risk of sexually transmitted infections even when with a monogamous partner). Future researchers could work with communities to identify appropriate sexual and substance use, including injection drug use, questions, to further contextualize HIV testing findings.

## 4 CONCLUSIONS

In conclusion, our study identified important socio‐demographic, social and structural factors associated with HIV testing among a gender‐diverse sample of TNB people. Findings suggest a need for trans‐inclusive HIV testing practices, including at the point of sexual violence intervention, and training PCPs in trans‐inclusion and gender affirmation. Ultimately, these interventions may increase the uptake of HIV testing among TNB people of diverse genders.

## COMPETING INTERESTS

The authors have no competing interests to disclose.

## AUTHORS’ CONTRIBUTIONS

ALD, SKK and AIS conceived of the study. LK and ALD completed data analyses, with consultation from AIS. ALD, SKK, FA and SY completed a first draft of the manuscript. All authors (ALD, LK, SKK, AIS, FA, SY and BAM) contributed to a second draft of the manuscript and approved the final version.

## FUNDING

The original MTHS study received no external funding. These analyses were partially supported by the National Institutes of Allergy and Infectious Diseases (NIAID) grant #1R03AI159298‐01.

## Data Availability

Data are available upon request to the University of Michigan Health Sciences and Behavioral Sciences Institutional Review Board (irbhsbs@umich.edu).
